# *Coquillettidia *(Culicidae, Diptera) mosquitoes are natural vectors of avian malaria in Africa

**DOI:** 10.1186/1475-2875-8-193

**Published:** 2009-08-10

**Authors:** Kevin Y Njabo, Anthony J Cornel, Ravinder NM Sehgal, Claire Loiseau, Wolfgang Buermann, Ryan J Harrigan, John Pollinger, Gediminas Valkiūnas, Thomas B Smith

**Affiliations:** 1 Center for Tropical Research, UCLA Institute of the Environment, Los Angeles, California, USA; 2 Mosquito Control and Biology, Kearney Agricultural Center, University of California, Davis, USA; 3 Department of Biology, San Francisco State University, CA 94132, USA; 4 Institute of Ecology Vilnius University Akademijos 2 Vilnius 2600, Lithuania

## Abstract

**Background:**

The mosquito vectors of *Plasmodium *spp. have largely been overlooked in studies of ecology and evolution of avian malaria and other vertebrates in wildlife.

**Methods:**

*Plasmodium *DNA from wild-caught *Coquillettidia *spp. collected from lowland forests in Cameroon was isolated and sequenced using nested PCR. Female *Coquillettidia aurites *were also dissected and salivary glands were isolated and microscopically examined for the presence of sporozoites.

**Results:**

In total, 33% (85/256) of mosquito pools tested positive for avian *Plasmodium *spp., harbouring at least eight distinct parasite lineages. Sporozoites of *Plasmodium *spp. were recorded in salivary glands of *C. aurites *supporting the PCR data that the parasites complete development in these mosquitoes. Results suggest *C. aurites*, *Coquillettidia pseudoconopas *and *Coquillettidia metallica *as new and important vectors of avian malaria in Africa. All parasite lineages recovered clustered with parasites formerly identified from several bird species and suggest the vectors capability of infecting birds from different families.

**Conclusion:**

Identifying the major vectors of avian *Plasmodium *spp. will assist in understanding the epizootiology of avian malaria, including differences in this disease distribution between pristine and disturbed landscapes.

## Background

Avian malaria parasites of the genus *Plasmodium *(Haemosporida, Plasmodiidae), are cosmopolitan mosquito-transmitted haematozoa [1,2]. In contrast to human malaria, avian malaria has a worldwide distribution and is caused by approximately 50 species of *Plasmodium *[3,4]. The widespread geographic distribution of avian malaria parasites and their broad range of avian hosts make them excellent models for exploring the ecological and evolutionary dynamics of vector-host-parasite associations.

Although some 50 species of avian *Plasmodium *have been identified using phenotypic characters [2,4], molecular data reveal remarkable genetic diversity of these parasites, indicating that the number of avian *Plasmodium *species and their taxonomic diversity may be greater than is accepted in the current classifications [5]. Avian *Plasmodium *are transmitted wherever the mosquitoes, susceptible birds and minimum temperature and humidity requirements are available. Many species of avian *Plasmodium *appear to have evolved with their hosts and do not typically cause lethal disease [6], although there are numerous reported cases of pathology and even high mortalities when naïve birds are exposed for the first time [6-8].

Although general patterns of the epizootiology of avian malaria have been well studied [9-11], little is known about the vectors and the parasites diversity within the vectors, an essential step in the avian malaria transmission cycle. Species within multiple mosquito genera (*Culex, Aedes, Culiseta, Anopheles, Mansonia *and *Aedeomyia*) have been implicated in the transmission of different species of avian *Plasmodium *[2,12-16]. Despite the presence of numerous ornithophilic species within the genus *Coquillettidia *only one report was found relating presence of sporozoites of *Plasmodium gallinaceum *and an unidentified *Plasmodium *sp. in the salivary glands of *Coquillettidia crassipes *[17]. Recently, DNA of a *Haemoproteus *species was isolated from 1/77 *Coquillettidia xanthogaster *collected in Vanuatu [18]; however, it is unclear if this parasite completes its life cycle in this mosquito.

In this study, the role of *Coquillettidia *spp. (Diptera, Culicidae) collected in the lowland forests of Cameroon as potential vectors of avian malarial parasites was explored. *Coquillettidia *is treated as a genus [19-21], rather than a subgenus of *Mansonia *[22-25]. Worldwide there are 57 described *Coquillettidia *species [21] with twenty-two from Africa. *Coquillettidia *spp. adults are medium in size and can easily be confused with *Aedes and Culex *mosquitoes, but all species (shared only with the genus *Mansonia*) have the unusual larval behaviour of attaching to air cells of aquatic plants to obtain oxygen for respiration [26]. Immature stages are mostly found in permanent bodies of water and only float to the surface as pupae just prior to eclosion. The integuments of most of the African species are bright yellow or have a yellow to greenish hue, which readily distinguishes them from other African mosquitoes [27]. Only one species, *Coquillettidia metallica*, has a dark body and legs [25].

During recent surveys of the lowland forests of Cameroon four species from the genus, (*Coquillettidia aurites, Coquillettidia pseudoconopas, C. metallica *and *Coquillettidia maculipennis*) were collected. These four species are endemic to Africa with known distributions in West, Central and East Africa [27,28]. They all are crepuscular [29,30], rarely bite man or other mammals [31] and prefer birds as hosts [32,33]. West Nile, Middleberg and Sindbis viruses have been isolated from *C. metallica*; while Tataguine, Simbu and Usutu viruses, closely related to West Nile and formerly recorded as this pathogen, have been isolated from *C. aurites *[32,34]. The viruses associated with *C. pseudoconopas *are not yet known [25]. Prior to this study, none of these species were implicated as vectors of malaria.

The avifauna of Cameroon is relatively well-studied and prior studies for blood parasites have identified *Plasmodium *spp. in many of the avian hosts [2,4,35-37], but little is known of the vectors.

The objectives of this paper were to: 1) use high throughput molecular genetic screening techniques of wild collected mosquito heads and thoraces to test for the presence of *Plasmodium *spp., 2) investigate the spatial distribution of the parasite mitochondrial cytochrome *b *(cyt *b*) sequence lineages found in *Coquillettidia *spp., 3) microscopically examine the salivary glands of the wild-caught mosquitoes for presence of sporozoites, which are the last stage of development of malaria parasites in vectors, and 4) compare the distribution and phylogenetic relationships of these lineages to published lineages found in other mosquito vectors and birds in Cameroon. This is the first molecular exploration of avian vector-avian host-malaria parasite relationships in Africa.

## Methods

### Sampling sites and habitat characterization

**Figure 1:**
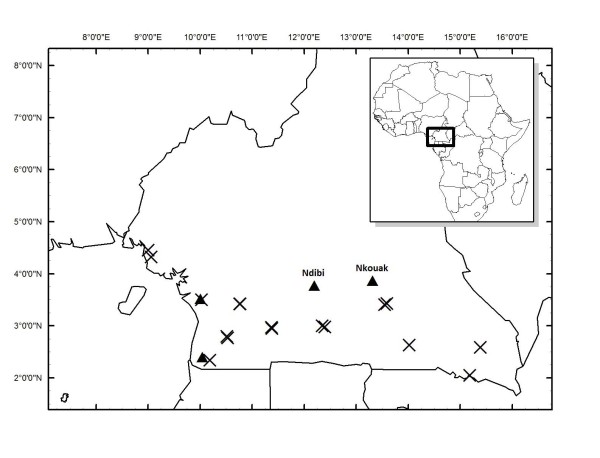
**Sampling locations of mosquitoes in the lowland forest areas of Cameroon**. Areas where *Coquillettidia *sp were recorded are indicated by filled triangles.

Sampling took place from June–August, 2007 and April–May 2008 at 20 sites in the lowland forests of Cameroon (Figure 1). *Coquillettidia *spp. were only found at four of those sites, Mvia (N03.5105° E010.0176°) Nk'leon (N02.3974° E010.0452°), Ndibi (N 03.774897° E12.201532°), and Nkouak (N03.86735° E013.31634°). The majority (95%) of the total numbers of *Coquillettidia *mosquitoes were collected in Ndibi and Nkouak. The lowland forests of southern Cameroon have a humid equatorial climate with three dry months characterized by alternating two dry seasons and two wet seasons (the big dry season between mid-November to mid-March, the small wet season from mid March to mid June, the small dry season from mid June to mid August, and the large wet season from mid August to mid November) [38]. The average annual temperature of the region is about 23°C (22.8°C in July to 24.6°C in April) [39].

Sampling was done at Mvia in June 11–13, 2007; Nk'leon in July 28–31, 2007; Ndibi, in August 21–26, 2007 and May 2–16, 2008; and Nkouak in August 27–31 2007 and again in May 17–29 2008. Nkouak is the most forested with the lowest human population density with fewer than 1000 people. The village is surrounded by swamps, which are the sources of the Nyong and Bomba rivers. Nkouak has three major vegetation types: (1) secondary forest immediately surrounding the village where locals have planted cocoa, coffee, some palms and other cash crops (2) mature secondary forest where very little human activity occurs, (3) mature forest, which is quite distant from the village and contain major wild life. Ndibi is characterized by secondary forest in various stages of degradation and seasonally flooded swamp forest (September to December) with floating grass plant communities along either side of the Nyong river [39]. Ndibi has a comparatively higher human population density and is less than 2 km from the city of Akonolinga (pop. 25, 700) across the River Nyong.

### Mosquito collections and preparation

Light trapping (mini CDC light traps) effort per site ranged from four to 15 trap nights. No notable changes in the weather occurred during the collection period that might bias comparisons of mosquito abundance.

**Figure 2:**
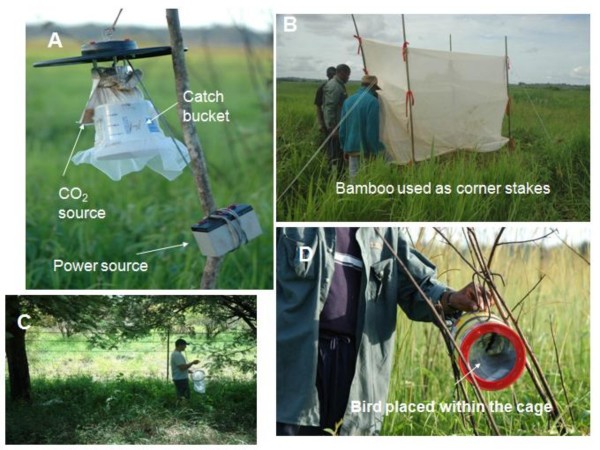
**Mosquito trapping methods used in this study**. A – CDC Light trap hung from dead tree in grassland along Nyong River, Ndibi; B – Net trap placed in grassland along Nyong River; C – Collecting mosquitoes resting in grass and on tree branches by sweep net. Mosquitoes were aspirated out from the sweep net and then placed into holding cages for identification and preservation. D – Ehrenberg bird trap hung in branches of dead tree in along edge of Nyong River grassland.

In 2007, mosquitoes were collected using six Center for Disease Control (CDC) Miniature Light Traps (Figure 2, [40]) baited with CO_2 _(John W. Hock, Gainesville, FL), following protocols for proper use and assembly provided with the traps. In the 2008 sampling season, a more comprehensive mosquito collection scheme was carried out in Ndibi and Nkouak that included use of six miniature CDC traps, four net traps (Figure 2, [41]), four modified bird baited Ehrenberg lard cans (Figure 2, [42]) and sweep net collections of resting mosquitoes in forest vegetation (Figure 2). To escape the heat and wind, mosquitoes rest in more humid conditions under leaves in shaded thick clumps of vegetation. At the collection sites, the surrounding forest offered many such resting sites. The use of CO_2 _(John W. Hock, Gainesville, FL) and birds (feral pigeons) as bait in the net traps was alternated. CO_2 _sachets and birds (confined within a cage) were placed in the middle of the net trap. In 2008 the lights from the CDC traps were switched off in order to reduce damage to mosquitoes (for morphological identification purposes) that would have been caused by thousands of captured moths and other arthropods attracted to light. In order to fully explore microhabitat preference and help broaden the sampling of the mosquito community, traps of each kind were placed along a vegetation gradient from the floating mat community, through the grass sedge meadow and upland forest and plantations to the swamp forest.

Traps were set out each day for at least 12 h (6.00 pm–06.00 am). Following each trapping period, the collection bags were removed from traps and the mosquitoes immobilized with chloroform and/or smoke within two hours of trap closing. On the same day following the collection and immobilization, mosquitoes were sorted by sex and identified to species with the aid of a stereomicroscope (× 90) and morphological keys [25,27]. Bird baited and CO_2 _baited traps collect predominantly blood-seeking females, and in many African mosquitoes, Culicidae closely related species can only be identified by examination of the male genitalia. This was the primary reason an attempt was made to find males in resting vegetation to identify the more cryptic species assuming that the males would give clues to identification of females collected in the baited traps. Sweep net collections were performed for an hour each day (3.00 pm–4.00 pm) in low-lying grasses and shrubs along the edges of foot paths and clearings in the forest floor. Females collected in the sweep nets were also used for *Plasmodium *spp. DNA isolation.

Collections were made at 20 sites, in the lowland forests of Cameroon, but trapping effort are analyzed for Ndibi and Nkouak only where more than 95% of *Coquillettidia *spp. were collected. Although resting mosquitoes were also collected, only mosquitoes collected overnight were used to calculate mosquitoes per trap night. For each site, sampling date, trap type and species, whole unfed and recently fed (blood meal still visible) mosquitoes were pooled separately into groups of between three to 20 individuals, and stored in 95% alcohol in the field and later at -20°C in the laboratory until DNA extraction and subsequent testing by PCR. Minimum field infection rates for estimating *Plasmodium *spp. infection per thousand pooled mosquitoes were calculated using the bias-corrected maximum likelihood estimation (MLE) methodology with 95% confidence skewness-corrected score intervals [43].

Chi-square analysis was applied to compare maximum likelihood infection rates (MLE) for all the *Coquillettidia *spp. and the number of isolated *Plasmodium *spp. infections as a function of the number of mosquitoes collected at each site. All statistical analyses were performed using STATA.

### DNA extraction, amplification and sequencing

In the laboratory the head and thorax of each mosquito was severed from the abdomens and pooled for DNA extraction. Pools varied in size from 3–20 mosquitoes, and DNA extraction followed modified procedures of Pilchart *et al *[44] and Ishtiaq *et al *[18] Briefly, sample pools were homogenized with the aid of heat-sealed pipette tips and 200 μL of total DNA was extracted using the DNeasy Tissue Kit (Qiagen) following the manufacturer's protocol except for the addition of 30 ml of 100 mg/ml dithiothreitol to the tissue digestion buffer [45]. The primer pairs L1518 and H15730 were used to amplify ~510 bp fragments of cyt *b *of the parasites. Each PCR cocktail contained 5.0 μL 10× Qiagen PCR buffer, pH 8.3 (10 mM Tris-CH1, pH 8.3 and 50 mM KC1, 0.01% NP-40), 1.5 mM MgCl_2_, 5 μL dNTP mix (10 mmol/L each), 0.3 μL Taq DNA polymerase (1.25 U/reaction), 2.5 μL each primer (0.1–0.5 μmol/L), 1 μL of 10% BSA buffer [46], 2 μL template DNA and the remaining volume of ddH_2_O up to 27.7 μL. The PCR thermal regime consisted of one cycle of 5 min at 95°C, 44 cycles of 0.5 min at 95°C, 0.5 min at 50°C, 0.45 min at 72°C, and a final cycle of 5 min at 72°C. Each extraction included a negative control (cocktail without the template DNA), which was screened to detect potential contamination, and a positive control. All positive pools were later subjected to nested PCR protocol [47]. In the first PCR reaction, the cyt *b *gene was amplified using primers HaemNF and HaemNR2 in a 25 μl volume reaction. For the second PCR, 2 μl of the first PCR product was used as template in a 25 μl volume with the primers HaemF and HaemR2, including the same reagents and under the same thermal regime as the first PCR, except that 35 cycles were used instead of 20 cycles.

Additionally, to ensure that the positive results were DNA from sporozoites and not from some undigested parasite infected blood cells that might have persisted in the vector digestive system (as remnants of blood meal), all positive samples were screened for vertebrate DNA using the same thermal regimes for the PCR reaction above, but with primers L14724 and H15149.

Finally, to help ensure that failure to detect a parasite was not due to poor DNA extractions, a fragment (650 bp) of vector COI DNA [48] was also amplified using primers LCO1490 and HCO2198 [49].

Positive or negative amplifications were evaluated as the presence or absence of bands on 1.5% agarose gels. Samples that showed positive amplification were subjected to dye terminator cycle sequencing reactions (30 cycles, 55°C annealing), and sequenced on ABI 3730 Genetic Analyzer (Applied Biosystems) automated sequencers, using Big Dye vs. 3.1. For all these samples, the mitochondrial cyt *b *gene was sequenced in two overlapping fragments using the same primer pairs described above.

Sequences were assembled with Sequencher 4.7 (Gene Codes Corporation, Ann Arbor, MI), aligned in ClustalX and manually corrected by eye. Because the sequences are from coding genes, alignment was straight-forward. Lastly, sequences were confirmed by BLASTN (basic local alignment search tool) to be most closely related to avian *Plasmodium *spp. cyt *b *(identical or nearly identical matches to lineages posted in GenBank). Potentially new and unique sequences were checked by additional sequencing of the fragments. The electropherograms were also checked for double nucleotide peaks to infer possible cases of mixed infections of two or more different parasite lineages. The new sequences have been deposited in the GenBank International Nucleotide Sequence Database (Accession numbers: GQ150187–GQ150196).

### Phylogenetic analysis

Phylogenetic analyses were carried out in PAUP* [50]. The compiled data set, excluding pools that showed indications of multiple infections, four additional species, *Plasmodium juxtanucleare, Plasmodium relictum, P. gallinaceum *and *Plasmodium elongatum*, all of which are known to be transmitted by other mosquitoes [2,16,17,51] and three outgroup species of positively identified avian haemoproteids, based on the phylogeny in Valkiūnas *et al *[4] was used. Additionally, four parasite lineages [36,52] recorded from numerous avian host species captured within the tropical lowland forests were included. Phylogenetic relationships of the parasite lineages were estimated using MrBayes 3.1 [53]. The most accurate available model of sequence evolution, the GTR + G model was used because this level of complexity is warranted due to the extreme AT bias in *Plasmodium *mitochondrial genes [54,55]. The Markov chain was sampled every 200 generations for 10 million generations. Bayesian posterior branch probabilities were obtained by taking the majority rule consensus of the sampled trees, excluding the first 12,500 trees as burnin. Replicate runs of the software, each with one cold and three heated chains, produced essentially identical results. Nodal support for the final tree was assessed using ML100 nonparametric bootstrap replicates each with TBR branch swapping. Additionally, PAUP* was used to estimate mean pairwise distances between the different *Plasmodium *lineages using the Kimura 2-parameter (K2P) model with uniform substitution rate among sites.

### Microscopic examination of salivary glands of mosquitoes

Nine wild-caught female *C. aurites *collected from Ndibi in February 2009 were dissected and salivary glands were isolated on glass slides using traditional mosquito dissection methods [56]. The head of insects was cut off with a razor and salivary glands were gently pressed out with a slight pressure by a blunt needle on the thorax near the base of the fore-legs. The glands were placed in a small drop of phosphate buffered saline, ruptured by a gentle pressure of a needle, and mixed with a minute drop of the saline to produce a thin film. The preparations were air-dried and fixed in absolute methanol in the field, and then stained with Giemsa in the laboratory, as described by Valkiūnas [2].

An Olympus BX61 light microscope equipped with Olympus DP70 digital camera and imaging software AnalySIS FIVE was used to examine slides, prepare illustrations and to take measurements. Entire films were examined at low magnification (× 400) and recorded sporozoites were studied at high magnification (× 1,000).

Representative preparations of sporozoites (accession numbers 47721, 47722 NS) have been deposited in the collection of the Institute of Ecology, Vilnius University, Vilnius, Lithuania.

## Results

### Mosquito collection

**Figure 3:**
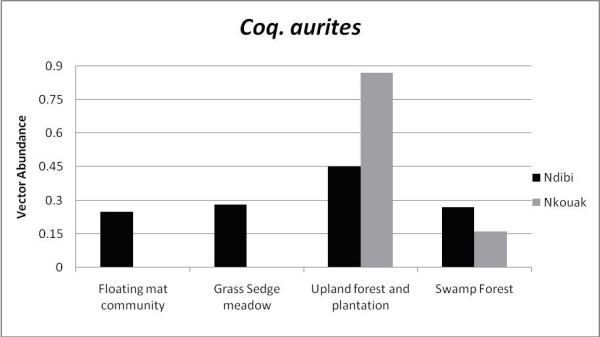
**Habitat preferences for *Coquillettidia aurites***. Overall, there was a significant preference for shaded areas (p = 0.04). No *C. pseudoconopas *was collected in Ndibi and *Plasmodium *DNA isolations were not associated with any of the four microhabitats. Collections from the other sites represent less than 5% of the total and are not included in the analysis.

In 2007 and 2008, 10,631 mosquitoes of at least 24 species were collected in Ndibi and Nkouak. Species of *Coquillettidia *accounted for 17% of the total mosquitoes collected (1,802/10,631) with over 76% captured with CDC light traps alone (Table 1). *Coquillettidia aurites *was by far the most common of the *Coquillettidia *species collected, comprising 71% of the species collected in Nkouak and only 9% of the total collected in Ndibi. *Coquillettidia metallica *accounted for 1.2% of the total, collected mostly in Ndibi and only a single blood fed individual was captured in Nkouak. *C. pseudoconopas *was captured in Nkouak, Mvia and Nk'leon and accounted for 15% of the total species collected (Table 1). Only one individual of *C. maculipennis *was captured in Ndibi during the entire season, so this species has been excluded from all the analyses. Relative population densities (light trap counts per trap night), for *C. aurites*, calculated for each site showed that Nkouak had a significantly higher number than Ndibi. The spatial geographic distribution of the *Coquillettidia *spp. mosquitoes as a function of habitat use is shown in Figure 3. Although no significant associations were demonstrated between the number of *Plasmodium *spp. isolations made from mosquitoes and any of the four microhabitats that were analyzed, most species showed (except for *C. metallica *that was mostly captured in open habitats) a strong preference for swamp forest/upland forest and plantations than more open habitats.

**Table 1:** Number of female *Coquillettidia *mosquitoes collected versus the four collecting modes in the two main sites, Ndibi and Nkouak

Species	CDC Light Traps (76.4%)	Net traps (9.4%)	Lard cans (12.8%)	Sweep net collections (1.3%)
*Coquillettidia aurites*	1147	156	196	12
*Coquillettidia pseudoconopas*	218	15	28	8
*Coquillettidia metallica*	11	0	7	3
*Coquillettidia maculipennis*	1	0	0	0

### Mosquito morphological considerations

Variation in dark apical scaling of sternites was found among female and male *C. aurites *with some having no dark scales, others with smattering of scales and in others dark scales formed complete apical bands. Males were collected resting in lower forest vegetation and their genitalia confirmed they were all *C. aurites *although the membranous expansion of gonostylus was not quite as large as that depicted in Service [25]. Images of male genitalia and other morphological features of mosquitoes are deposited in [57].

### Variation in malaria prevalence among *Coquillettidia *species

**Figure 4:**
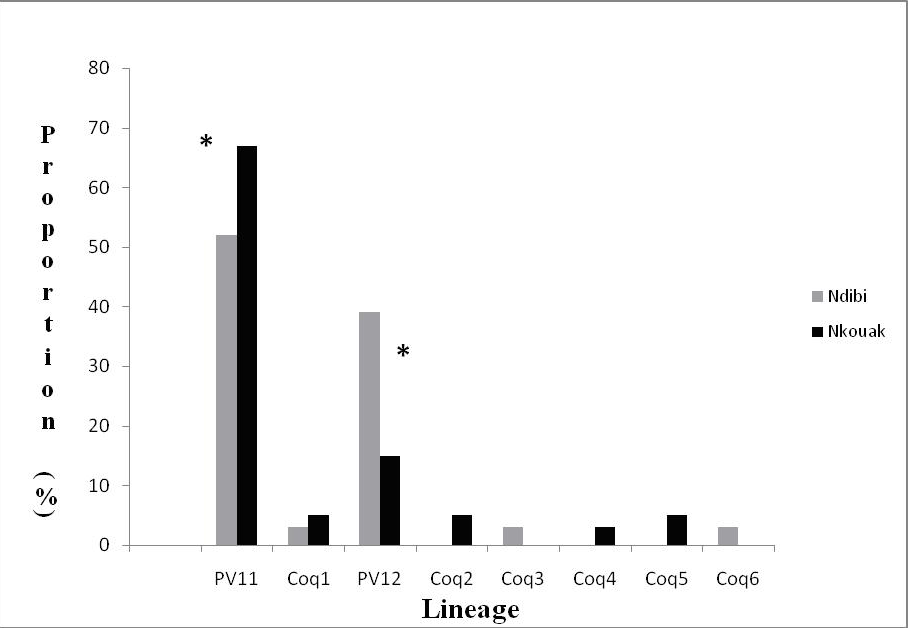
**Proportion of infected pools of the eight *Plasmodium *lineages of *Coquillettidia *sppin Ndibi and Nkouak**. Because sites sampled are more than 100 km apart, populations are considered independent. * indicates significant differences in proportion of infected pools between both habitats. Lineages PV11 and PV12 were the most common while the others were relatively rare.

The number of positive pools in the three species is presented in Table 2. No vertebrate DNA was found in any of the pools, which shows that only DNA of *Plasmodium *spp. sporozoites (but not of blood stages of the parasites) was amplified. Of the 256 (1285 individuals total) pools screened for *Plasmodium *spp., 85 (33%) were positive for parasites, yielding a maximum likelihood estimates (MLE) of *Plasmodium *infections of 112.22/1,000 for *C. metallica*, 84.04/1,000 for *C. aurites *and 35.4/1,000 for *C. pseudoconopas*. Within the two sites however, *C. aurites *showed a significantly higher MLE in Ndibi than in Nkouak (Ndibi = 116.46/1000; Nkouak = 66.76/1000; χ^2 ^= 13.49, p < 0.001), although Nkouak had a higher density of *C. aurites *(Figure 4). Average mosquito densities within the sites ranged from 39.8 mosquito/trap-night in Ndibi to 115 mosquito/trap-night in Nkouak, whereas the average proportion of corresponding *Plasmodium *isolations ranged from 45.12% in Ndibi to 28.99% in Nkouak.

**Table 2:** Proportion of vectors pools that were screened by PCR for malaria parasites by species.

	PCR Screen					
				
Species	Negative	Positive	Total	Proportion positive	MLE/1000	Number of individuals
						
*Coquillettidia aurites*	143	77	220	0.35	84.04	1082
*Coquillettidia pseudoconopas*	25	6	31	0.19	35.54	183
*Coquillettidia metallica*	3	2	5	0.4	112.22	20
Total	173	85	256			1285

MLE represents Maximum likelihood estimates of infection rates

### Distribution of *Plasmodium *spp. lineages among *Coquillettidia *spp

Parasite lineages based on 750 nucleotides of cyt *b *sequence were distinguished after checking carefully against the original chromatograms from the sequencing gel. Parasite sequences differing by only a single nucleotide were considered the same phylogenetic species, while numbered lineages differing by more than one nucleotide substitutions, or at least 0.2% genetic distance were considered separate phylogenetic species. At least eight lineages of *Plasmodium *spp. were found from the positive pools. Two of them were already known lineages (PV11 and PV12) [36] and six were new lineages that were named by assigning a number in sequential order (PlasCoq1- PlasCoq6). The 8 lineages differed from one another by 0.2 and 15.8% sequence divergence. Some of these are small genetic distances, and likely represent intraspecies divergence of avian *Plasmodium *[4] although in some cases they are well beyond distances corresponding to good species [5]. Cytochrome *b *sequence divergence as low as 1.0% [58] has been observed between named species of malaria parasites of mammals, although biological 'species' boundaries of avian malarial parasite lineages have not been clearly defined. Of the eight *Plasmodium *lineages found in this genus, *C. aurites *harboured seven lineages; *C. metallica *at least two (including an additional co- infection which was not included in the analysis) and *C. pseudoconopas *harboured one. The most common lineages (PV11 and PV12) were found to be unevenly distributed in both regions (Figure 4) and represent more than 88% of the lineages recovered from these mosquitoes. The other lineages were found in very few mosquitoes in both locations (Figure 4). Lineage PlasCoq6 is widely divergent from the other lineages, and was only recorded once in a pool sampled from Ndibi. These rare lineages were re-sampled.

### Microscopic examination of salivary glands of mosquitoes

**Figure 5:**
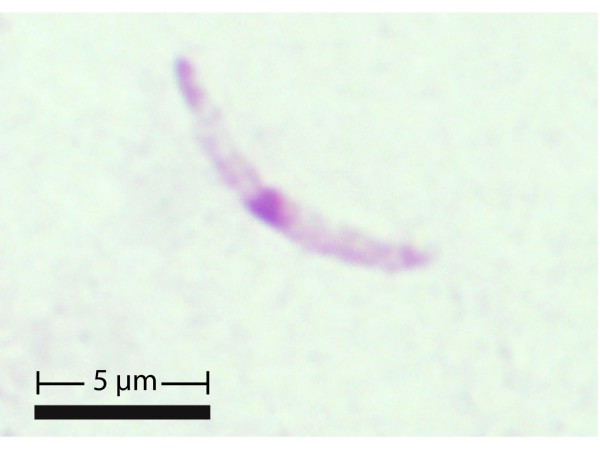
**Giemsa-stained sporozoite of *Plasmodium *spp. from the salivary glands of *Coquillettidia aurites***. Note a prominent centrally located nucleus. (Scale bar = 5 μm).

Sporozoites were observed in salivary glands of two mosquitoes. Up to three sporozoites were observed in each positive salivary gland smear. They were of elongated shape typical for haemosporidian parasites, with nuclei located approximately at the center (Figure 5). Non-deformed sporozoites (n = 3) averaged 9.9 μm in length and 0.9 μm in width.

### Distribution of lineages among avian hosts

**Figure 6:**
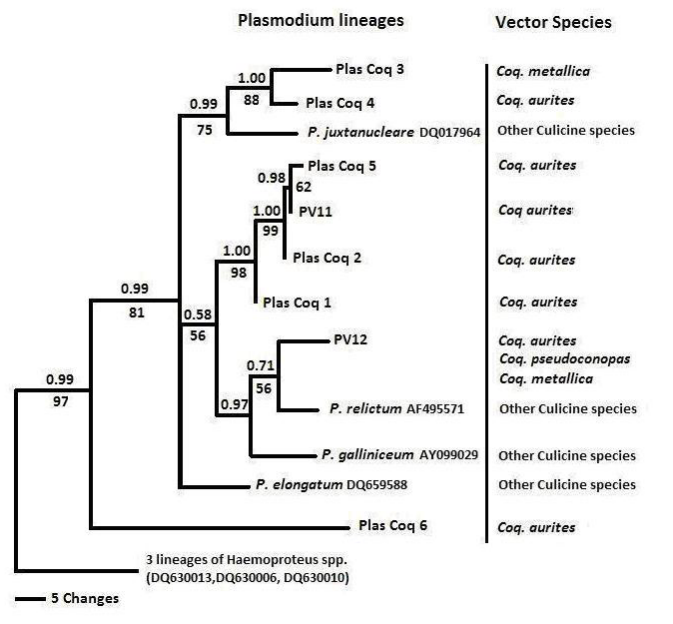
**Bayesian phylogeny of the 8 lineages of *Plasmodium *mitochondrial cytochrome *b *gene obtained from *Coquillettidia *species, 4 lineages from published sequences of avian *Plasmodium *and three lineages of *Haemoproteus *spp. as outgroups**. Names of the lineages and GenBank accession numbers of the sequences are given after the species names of parasites. Bayesian support are indicated above the branches while ML Bootstrap support, based on 100 replications are shown below the branches. The vector species in which the parasites were found (including parasites already known) is indicated under 'Vector Species.'

Identical lineages identified based on sequence similarity as posted on GenBank were found in parasites isolated from birds belonging to different families of the Passeriformes and non Passeriformes by Blastn search. PV11 [36] is an identical match to WA15 [52], while PV12 [36] is identical to lineages WA9 and P46 [3,52]. Over 35 species of birds from 11 families have been shown to be infected with these lineages. The other lineages were relatively rare and together comprised less than 12% of the total parasite load in *Coquillettidia *spp. Lineages PlasCoq2 and PlasCoq5 however showed a close relationship to PV11 (Figure 6). In general, the parasites obtained from *Coquillettidia *spp. consisted of closely related lineages also found in birds of different families and these lineages do not appear to be shared with lineages found in other mosquito species so far (Figure 6). While further sampling will be necessary, it is possible that these parasites are only transmitted by *Coquillettidia *spp.

### Variation in parasite prevalence with respect to vector abundance

The likelihood estimates of infection rate (MLE) was examined as a function of the sample size of vectors by asking whether less common species had different infection prevalence than more common species, assuming that species were captured in CDC traps in proportion to their relative abundance. Interestingly, the least and most abundantly sampled species, *C. metallica *and *C. aurites *respectively, had the highest maximum likelihood estimates of infection rates (Table 2). Because only few *C. metallica *were collected, it was not possible to determine if these high estimates of the likelihood infection rates truly reflect the infection rates in nature. More sampling is warranted to confirm this.

### Phylogenetic analysis and distribution of *Plasmodium *spp. cytochrome b lineages in *Coquillettidia *spp

Figure 6 summarizes the most important phylogenetic results of this study. Of the 85 pools infected with *Plasmodium *spp., 81 yielded sequence data of sufficient length to be included in the phylogenetic analysis. The remaining cases (n = 4), showed indications of multiple infections as revealed by double peaks in the chromatogram, and were not included in the analyses. However, some double peaks were easily discernable for parasites lineages PV11 and PV12. All parasites were designated as avian *Plasmodium *spp. by phylogenetic affinity to published avian *Plasmodium *spp. cyt *b *sequences. Support for the nodes, judged by the bootstrap analysis is strong. Examination of the tree revealed no reciprocal monophyly of the *Plasmodium *spp. lineages detected in *Coquillettidia *spp., but they clustered together as separate clades with lineages recovered from other culicine species nested within the tree. Known avian parasites, *P. relictum *and *P. elongatum*, both of which are transmitted by other vector *s*pecies [16,51] tend to show close affinity to lineage PV12. *P. juxtanucleare *was most closely related to lineages PlasCoq3 and PlasCoq4. Lineage PlasCoq6 was very rare and was only found once, and this lineage is basal to the tree and equally distant to *Plasmodium *and *Haemoproteus*.

## Discussion

Dissection of the midguts and salivary glands of mosquitoes, with subsequent microscopic examination of preparations of ookinetes, oocysts and sporozoites, have traditionally been used for the detection of malaria parasites in vectors [56,59-61]. These techniques, although sensitive, are laborious, and demand fresh material. The PCR procedure is sensitive enough to detect as few as 10 sporozoites in salivary glands and presence of a single oocyst is easily identified by simple agarose gel analysis [18], so this is a promising method for investigation of avian malaria vectors.

Mosquitoes utilize a number of cues to first locate, and then accept or reject a potential host [62,63]. A general cue like CO_2 _may elicit host-seeking behaviour in a wide-range of host specific mosquito species like *Coquillettidia *spp. regardless of the source. However, when a mosquito locates a potential host through long-range broad-based cues, another set of highly specific short-range cues ultimately lead to host acceptance or rejection. These results show that CO_2 _was an effective cue for attracting *Coquillettidia *spp.

These results also show that the avian *Plasmodium *parasites can be consistently isolated from wild caught *Coquillettidia *spp. mosquitoes confirming some *Coquillettidia *species as newly recognized major vectors for avian malaria assuming these mosquitoes are susceptible at least to some of the detected *Plasmodium *spp. lineages. Because 1) sporozoites were detected in salivary glands of *Coquillettidia *spp., and 2) only *Plasmodium *spp. complete sporogony in mosquitoes [2], it is unlikely that the sporozoites recorded in salivary gland (Figure 5) might belong to haemosporidian parasites belonging to other genera. The size of the sporozoites coincides with the size of sporozoites of *Plasmodium (Novyella) rouxi*, a widespread malaria parasite of passeriform birds [56]. To prove that viable sporozoites develop in *Coquillettidia *spp., experimental infections of susceptible avian hosts are needed. Interestingly, all the parasite lineages recovered from *Coquillettidia *spp. clustered with parasites identified from several bird species suggesting the vector's potential of feeding on and infecting birds belonging to different families. These results therefore implicitly confirm the mostly ornithophilic behaviour of these mosquitoes, and provide a genetic link between the infected birds and the mosquito vector, thus defining a complete transmission cycle. A more conclusive statement to determine mosquito-feeding specificity could be done by determining vertebrate DNA in fed mosquitoes. The present study thus provides clear directions for further experimental research on vectors of avian malaria in Africa.

### Vector-parasite-avian host association

This study found that over 90% of the pools of *Coquillettidia *spp. that were positive consistently harboured certain parasite lineages, which were closely associated to lineages isolated from different bird host groups. All the positive pools were screened for vertebrate DNA to confirm that none had contaminant bird DNA, and the absence of vertebrate DNA from all pools suggests that *Plasmodium *spp. lineages were isolated from sporozoites rather than amplified from blood stages of the parasites. At least one previous study has suggested that *Coquillettidia crassipes *is a natural vector of *P. gallinaceum *and another unidentified *Plasmodium *sp. in Ceylon [17].

Recently, several new species and lineages of malaria parasites of the subgenus *Novyella *have been described from African birds, *Cyanomitra olivaceae *and *Andropadus latirostris *from the same study sites where *Coquillettidia *spp. were collected [4,37]. Interestingly, lineages of these parasites were not found in *Coquillettidia *spp. It is thus probable that these lineages may not be transmitted by *Coquillettidia *spp., but that *Coquillettidia *spp. may be important vectors of eight other *Plasmodium *spp. lineages described in the present study. Species identities of these lineages remain unknown. It is not unprecedented for a malaria parasite lineage to be restricted to infecting limited species within just one mosquito genus (but see Valkiūnas [2] for avian malaria). For instance, five species of anopheline mosquitoes serve as the major vectors for *P. falciparum *in continental sub-Saharan Africa, and all five species belong to the subgenus *Cellia *[64-68]. Such strict vector specificity may however, not hold true for avian malaria since these bird parasites are very diverse. In avian malarial systems, the vectors of *Plasmodium forresteri*, *P. elongatum*, *P. juxtanucleare, P. gallinaceum*, and *Plasmodium kempi *do not belong to same genus [16,51,69]. If avian *Plasmodium *spp. lineages seem to a large extend to be host-generalists [5,59,70-72], one would expect expansion of parasites to occur freely if the appropriate vectors are present.

### *Plasmodium *spp. prevalence and diversity

At least eight *Plasmodium *spp. lineages were found in this study, with two of the lineages, PV11 and PV12 being the most common, occurring in over 88% of the positive pools screened. The reason for the existence of multiple distinct *Plasmodium *lineages found in mosquitoes of the genus *Coquillettidia *(seven of which are found in *C. aurites *alone) is not known, but may provide a plausible explanation for the co-occurrence of two or more parasite lineages in the same vertebrate host [36] and clarify the contrasting views of Oaks *et al *[64] and Gager *et al *[73]. The former purported that multiple parasite species that share a vertebrate host could be transmitted by the same mosquitoes genera or species, while the latter's explanation for co-occurrence of congeneric malarial parasites in an avian host to transmission by mosquitoes of different genera. Further work with multiple independent markers from different parts of the *Plasmodium *spp. genome should be carried out to clarify species limits.

The genetic distance between recorded lineages ranged between 0.2% and 17.4%. Because genetic differences between lineages PV11, PlasCoq2 and PasCoq5 are small (Figure 6), it is possible that these three lineages represent genetic variations of the same species, as has been shown to be the case with numerous lineages of avian *P. relictum *and several avian *Haemoproteus *spp. [4,37]. Lineages with genetic distances of <5% in cytochrome *b *gene has been shown to be a measure representing intraspecific variation in avian *Plasmodium *spp, although there are exceptions to this rule [4]. Thus, the molecular criterion of > 5% sequence divergence in cytochrome *b *gene for identification of haemosporidian species should be applied only through the careful linkage of molecular and microscopical data. Some of the genetic distances observed in this study are within this range, and higher than the average pairwise distances between *Plasmodium *cytochrome *b *sequences from many other avian malaria studies [74], as well as the average cytochrome *b *distance among three most closely related human malarial parasites that are known to be different species, i.e. *Plasmodium vivax*, *Plasmodium malariae *and *Plasmodium ovale *[58].

## Conclusion

This study strongly suggests that *Coquillettidia *spp. are vectors of avian malaria in African rainforests. Six previously unknown lineages of avian *Plasmodium *parasites were uncovered, and high numbers of *Plasmodium *lineages in a single vector species were detected; that includes the previously unknown lineages and two known lineages PV11 and PV12. The high diversity of *Plasmodium *parasites in *Coquillettidia *spp. is unprecedented among ornithophilic mosquitoes. Whether this diversity is due to their abundance in areas where they occur, their competitive or immuno-competent abilities, or a combination of these and other factors remains to be understood. Any link between these factors and the success of *Coquillettidia *spp. in anthropogenic landscapes would be greatly informative to effective vector control and avian malaria epidemiology studies.

## Competing interests

The authors declare that they have no competing interests.

## Authors' contributions

KYN designed the study, collected the field samples, performed nested PCR and Sequencing reactions, the statistical analysis and drafted the manuscript. AJC collected the field samples, did morphological identifications of specimens and helped in data analysis. RNMS and CL participated in the design of the study, and helped with nested PCR and data analysis. RJH, WB and JP participated in the design of the study and helped in data analysis. GV identified the sporozoites from the salivary glands and helped in data analysis. TBS conceived of the study, participated in its design and coordination and helped in writing the manuscript. All authors have read and approved the final manuscript.
